# Supplementary figures and images for: Measuring constitutional preferences: A new method for analyzing public consultation data
Source: PLoS One. 2023 Dec 14;18(12):e0295396. doi: 10.1371/journal.pone.0295396 (PMC10721082; doi:10.1371/journal.pone.0295396)

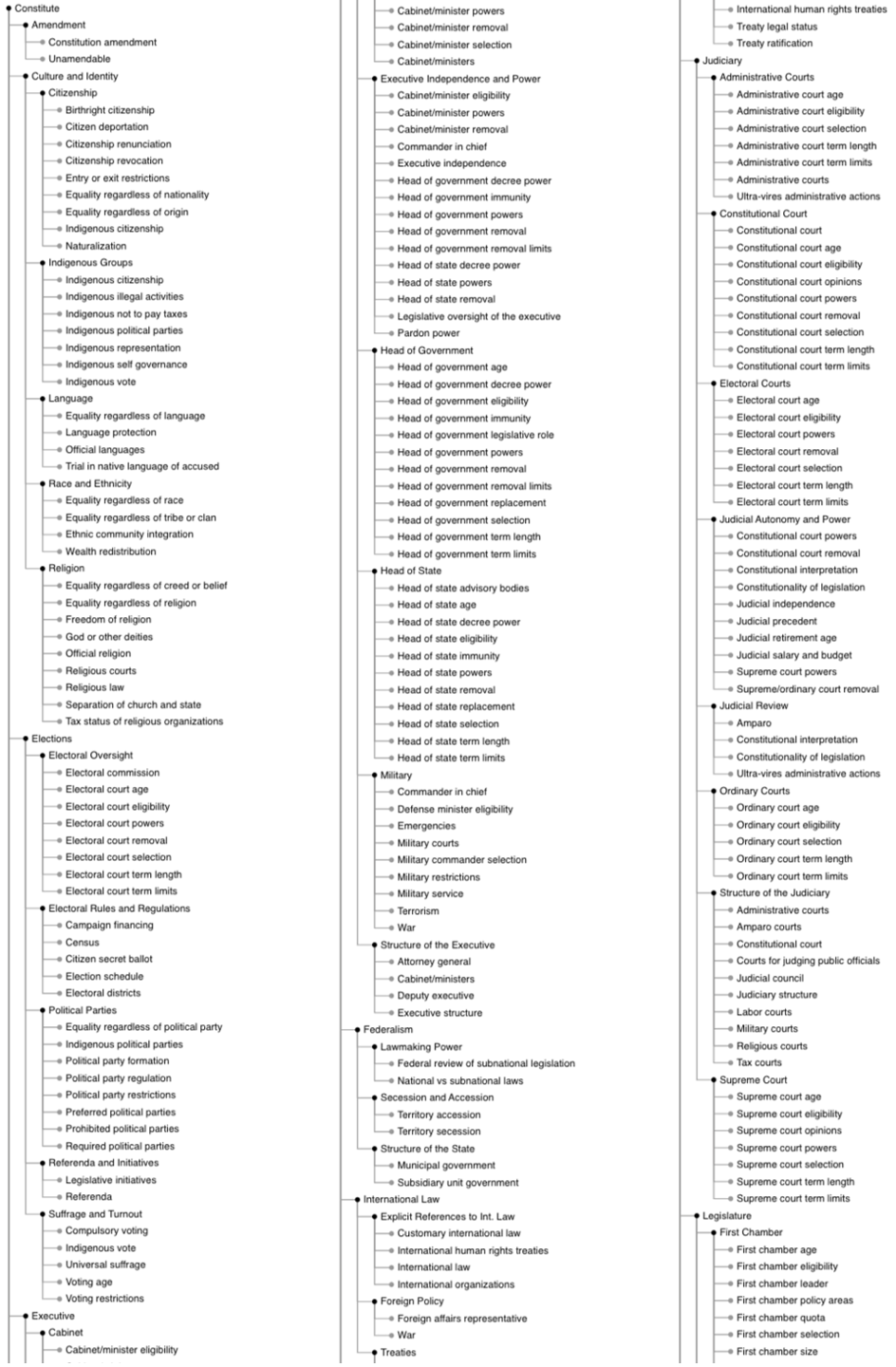

Supplement: S1 Appendix — Below is a representation of the 334 topics in the CCP ontology, organized into 12 categories. (TIF) [file pone.0295396.s001.tif]

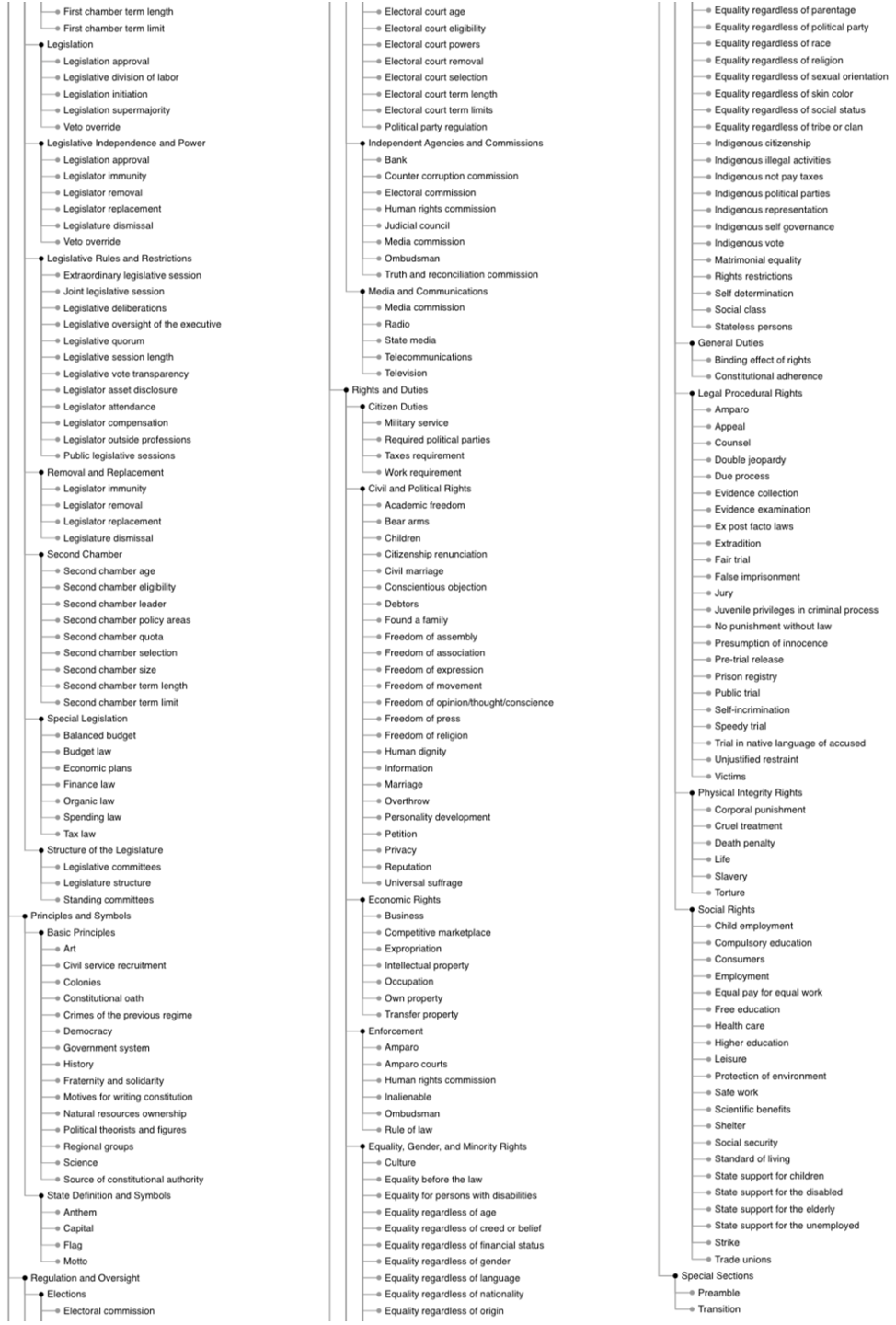

Supplement: S2 Appendix — The repository contains the following folders and files to support replication of our results in this paper: data (CCP ontology, CCP institution topics, CCP rights topics, municipality types, and predefined consultation topics), outputs (distribution data and municipality data that are calculated spreadsheets containing counts, scaled values, and percentages generated from the main similarity scores and used in data analysis), proc (main similarity scores), Jupyter notebook used to build calculated spreadsheets from the main similarity scores, and Jupyter notebook used for data analysis. (TIF) [file pone.0295396.s002.tif]
