# Supplementary material for: Measuring constitutional preferences: A new method for analyzing public consultation data
Source: PLoS One. 2023 Dec 14;18(12):e0295396. doi: 10.1371/journal.pone.0295396 (PMC10721082; doi:10.1371/journal.pone.0295396)
Supplement: Attachment — Submitted filename: Response to reviewers.docx [file pone.0295396.s003.docx]

Response to reviewers

We thoroughly appreciate the detailed and helpful comments received from the editor and reviewers. We note below how we sought to address each comment and where those changes can be found in the manuscript.

## Editor comments

- Clarify what exactly you are measuring here and how policy implications depend on what your data reflects. Do you measure knowledge of constitutions and what topics they typically deal with? Do you measure what topics are particularly salient in the public debate? Are you measuring actual priorities in the design process as perceived by citizens? To what extent can we really infer from your measures "what the people want", as you claim in your title?

In the Introduction and Materials and Methods sections, we clarified that we are measuring the constitutional preferences of Chilean participants by comparing them to the topics in all current national constitutions. In the Summary, we clarified the specific findings—and thus the scope of policy implications—related to each measure.

We significantly reworked the structure of the Results section to improve the narrative flow and reinforce our measurement approach, in particular to: 1) explain the justification for using aggregated response data in the constitution-consultation comparisons, 2) simplify tables of correlations, 3) remove the reporting of correlations between constitutions and administrative levels and municipality types, and 4) integrate tables of correlation with the figures for all topics, rights topics, and institutional topics.

- Make sure that notes on all figures and tables clarify what exactly they represent without consulting the text.

Throughout the paper, we expanded the captions on all figures and tables.

- Report information about statistical significance tests consistently and correctly.

In the Results section, we simplified the tabular presentation of correlations and quoted p-value thresholds.

## Reviewer 1

- Is there a strategy / way forward for generalizing this / incorporating more cases so that claims can be made about this alignment generally as opposed to just the Chilean case?

In the Conclusion, we added a final paragraph to convey how we are actively applying the methodology to other corpora.

- What about the use of covariates to facilitate testing of hypotheses about why there is / isn’t alignment? This has been attempted before and should not be difficult to implement in topic modeling packages. Or at least articulation of hypotheses for future research?

In our applied example, we examine two possible covariates: consultation structure and residential context. Our technique of conceptual alignment lends itself nicely to any ex post analysis of covariates—the reference ontology introduces an unmovable base that facilitates comparisons across different crosscuts of a corpus (compare this to the more unstable approach of feeding covariates ex ante to a topic modeling algorithm). We have added a snippet to the Conclusion mentioning the flexibility of this approach in introducing and analyzing any available covariates for a given corpus.

- Conclusion 1, Chilean interest isn’t the same as global interest: can you really say this given (as acknowledged earlier) that the public was guided by prompts from the drafters?

In conclusion 1 in the Results Summary, we more clearly articulated the influence of predefined concepts and other factors that shaped Chilean responses—and why the finding remains of interest to constitution drafters.

- P. 29, same problem: can you really say Chileans disproportionately focused on rights, given the drafters’ prompts? You can say they focused disproportionately on equality rights, OK, but rights generally?

In the Discussion, we clarified that it is the consultation organizers who focused disproportionately on rights in how they structured the consultation. We also added a paragraph highlighting consultation participants’ strong focus on equality issues within the scope of rights issues raised.

## Reviewer 2

1. The research design of the study can be improved substantially:
   a. To enhance the quality of this study, authors should refine the research questions. This refinement will greatly assist the authors in establishing a distinct theoretical framework and a robust empirical strategy. I strongly recommend concentrating on either the questions outlined within the "consultation structure" or those in "residential context" sections. It is advisable to refrain from making any causal claims due to the limitations of the available data, as asserting any form of causality based on the existing data is not feasible.

We reframed the research questions to clearly state the aims of the administrative level and municipality type analyses. This is also reflected in the updated Results section which clarifies the use of administrative level and municipality type analyses to justify the use of aggregated response data. No causal claims are made in the paper.

b. The current work lacks a citation of relevant literature on the text-as-data approach within legal scholarship. To address this, the authors could consider starting their exploration with the comprehensive study conducted by Frankenreiter and Livermore (2020), which offers an insightful survey of computational methodologies in the realm of law. This survey notably encompasses research close to the present study in terms of substantive content. Furthermore, it is advisable for the authors to actively seek out recent studies that bear resemblance to their research in terms of methodology, such as the work by Nyarko (2019).

Our Discussion section contains a review of NLP methods to highlight the advantages of the sentence embedding technology we use. Given this is primarily a methodological paper on an application of this technology, we feel a more general literature review of a field as diverse as text-as-data would be inappropriate. We do not see the work of Nyarko (2019) as bearing resemblance to our work. Nyarko trains a Bayesian classifier and uses word embeddings to identify and correct misspellings, whereas our work uses sentence-level embeddings to identify and compare semantically similar text segments across multiple corpora in multiple languages. While the Frankenreiter and Livermore review provides a useful overview of broader computational methods, we also don’t see this as being applicable for inclusion in citations related to our narrower focus on developing a new methodology for applying sentence embedding technology. The Frankenreiter and Livermore review includes two citations related to word and document embeddings on page 44, but neither use sentence embeddings.

c. The paper is deficient in terms of a coherent theoretical framework. While various concepts are introduced, they are presented without robust substantiation. This situation could be a result of absence of a well-defined research question. It is strongly advisable for the authors to rework and clarify their research question, subsequently constructing a theoretical framework that can serve as a foundation for generating empirically testable hypotheses.

We see this paper as making a methodological, rather than a theoretical, contribution by presenting a methodology that can be used to generate research questions and testable hypotheses. We thus seek to demonstrate the viability of the methodology by testing some of our research questions. To address the reviewer’s comment related to research questions, we clarified our research questions in terms of the substantive and methodological components they entail (in the Materials and Methods section) and more clearly tied this to our findings (in the Results Summary).

2. The precise assessment of the empirical strategy employed in this study is impossible due to the absence of well-defined hypotheses for investigation.

As noted above, we see the central goal of this paper as presenting a methodology that can be used to generate research questions and testable hypotheses. We thus seek to show the viability of the methodology by testing some of our research questions.

a. The rationale behind the selection of global constitutions as a basis for comparison remains unexplained. A potentially more justifiable approach could involve examining consultations across distinct geographical regions and varying levels of engagement. As it stands, the current empirical evidence is notably disorderly and fails to yield any novel insights.

Regarding the reviewer’s comment on global constitutions as the comparison point: In the Introduction and Materials and Methods sections, we underscored that global constitutions provide a useful point of comparison to highlight both what is and what isn’t raised in consultation responses on constitutional content. In the Conclusion, we also noted that the global constitutional corpus and ontology we used is just one of potentially many corpora and ontologies that could be used with this method. Regarding the reviewer’s comment on empirical evidence: In the Results Summary, we clarified the specific findings produced by each of our measures to convey the novel insights produced by our methodology.

b. The assertion regarding the paper's methodological contribution is inaccurate. The methodology used in the paper was originally introduced by Yang et al. in 2019.

We are aware of the Yang et al. 2019 paper and its omission was a mistake. It has now been added and cited in the paper. However, notice that our methodological contribution is different from theirs. In tandem, the Cer et al. (2017) and Yang et al. (2019) pieces introduce the USE model for multilingual sentence embeddings that we employ. Our methodological innovation is in applying their model in a way that facilitates the digestion of a complex collection of text. We show how sentence-level embeddings can be used to align an unwieldy corpus with a more manageable reference ontology, greatly improving interpretability.

c. Considering the swift progression in the field of large language models, it becomes apparent that the model employed in this paper is not the most current iteration available.

The model we use is Google’s current multilingual model for text classification, text clustering, semantic textual similarity retrieval, and cross-lingual text retrieval. Our choice of model was determined by performance in standard benchmarks, speed of computation, and an open-source license. The model is portable and can be deployed on our own machines. Note, however, that our methodology is based on the ability to perform sentence-level semantic similarity and is independent of the model generating the embeddings. Assessment of other models is on-going and if a better model is found that meets our selection criteria then it would be adopted.

d. Even granting the assumption that the model used in this paper is of high quality, a pressing question arises: why is this specific model suited for the given task? The authors neglect to address this inquiry satisfactorily.

The model produces sentence-level embeddings and supports the measurement of semantic similarity. The advantages of this type of model are addressed in the Discussion section. Importantly, the model is multilingual—a requirement when comparing English topics to consultation responses in Spanish, which we noted in the Methodology section.

e. They omit a comparative analysis with other existing models and fail to present a theoretical rationale for the efficacy of their chosen model for the task at hand.

See response to point c above. As noted, the central point is that our methodology is based on the ability to perform sentence-level semantic similarity and is independent of the model. We use the best-performing model currently available—the performance of which is discussed in Yang et al. (2019).

f. Furthermore, the authors provide no validation for the reliability of their dataset.

If dataset refers to the constitutional and consultation textual data, then these are complete datasets not samples and reliability is not an issue. If dataset refers to the semantic similarity measures between topics and constitution sections and consultation responses, these measures are objective and replicable. The replication data are provided at the url included in Appendix B.

3. The language used in the paper can be improved substantially.
   a. Many sentences are ambiguous and unclear. Examples:
      i. The country’s 2016 consultation process was similar to that of other contemporary constitutional projects and may be something of a model case, given the regional influence of Chile and the historic nature of its reform.

We sought to clarify any ambiguities in language throughout the paper.

b. Instead of relevant terminology, new concepts were developed. Examples:
   i. Constitutional Ontology instead of ground truth.

The ontology is not a ground truth dataset. The CCP ontology we use is a set of concepts and categories developed to systematically track the content of constitutions. We use the topic labels and descriptions in this ontology as the set of sentence-level phrases we use to retrieve semantically similar sentence-level text from constitutions and consultation responses. To address the reviewer’s comment, we clarified this in the Data section on the reference ontology.

## Reviewer 3

- Maybe give a bit more context on the Chilean process. E.g., gender equality is one of the main topics in public consultation, but it was also the first constituent assembly with gender parity.

In the Data section, we added a paragraph on the context of the consultation process that produced the consultation data we use as one of our textual data sources. In the Discussion, we added reference to the unique composition of the constitutional convention, particularly in terms of gender parity.

- More generally, some reflection on why equality is so high on the agenda would be useful. Maybe use the discussion for some of that? Overall, a very useful contribution to the literature.

In the Discussion, we added a paragraph highlighting the strong focus on equality issues in the consultation responses.
